# BRCA1 and Its Network of Interacting Partners 

**DOI:** 10.3390/biology2010040

**Published:** 2012-12-31

**Authors:** Charita M. Christou, Kyriacos Kyriacou

**Affiliations:** Department of Electron Microscopy/Molecular Pathology, Cyprus School of Molecular Medicine, The Cyprus Institute of Neurology and Genetics, 6 International Airport Avenue, P.O. Box 23462, 1683 Nicosia, Cyprus; E-Mail: kyriacos@cing.ac.cy

**Keywords:** BRCA1, DNA repair damage response, HR, cell cycle checkpoint regulation, phosphorylation, E3 ligase, ubiquitination

## Abstract

BRCA1 is a large multi-domain protein with a pivotal role in maintaining genome stability and cell cycle progression. Germline mutations in the *BRCA1* gene confer an estimated lifetime risk of 60%–80% for breast cancer and 15%–60% for ovarian cancer. Many of the germline mutations associated with cancer development are concentrated in the amino terminal RING domain and the carboxyl terminal BRCT motifs of BRCA1, which are the most well-characterized regions of the protein. The function of BRCA1 in DNA repair, transcription and cell cycle control through the DNA damage response is orchestrated through its association with an impressive repertoire of protein complexes. The association of BRCA1 with ATM/ATR, CHK2 and Aurora A protein kinases regulates cell cycle progression, whilst its association with RAD51 has a direct impact on the repair of double strand DNA breaks (DSBs) by homologous recombination (HR). BRCA1 interactions with the MRN complex of proteins, with the BRCC complex of proteins that exhibit E3 ligase activity and with the phosphor proteins CtIP, BACH1 (BRIP1) and Abraxas (CCDC98) are also implicated in DNA repair mechanisms and cell cycle checkpoint control. BRCA1 through its association with specific proteins and multi-protein complexes is a sentinel of the normal cell cycle control and DNA repair.

## 1. Introduction

*BRCA1* is a well known tumor suppressor gene identified in the nineties and germline mutations in this gene confer increased susceptibility to developing breast and ovarian cancer [1,2]. Its discovery and association with cancer development sparked a flurry of numerous studies investigating the functions of this protein. It is now well established that BRCA1 is involved in DNA repair by HR, cell cycle checkpoint regulation, transcription and apoptosis [3,4,5,6,7,8]. However, it is believed that the entire spectrum of BRCA1 activities remains to be discovered and that its tumor suppression function is yet to be defined.

**Figure 1:**
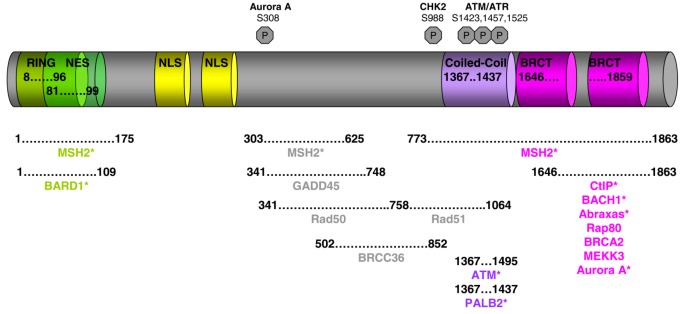
BRCA1 functional domains and its partners. Asterisks denote direct interaction with BRCA1.

The *BRCA1* gene is located on the long arm of chromosome 17 [9]. It comprises 24 exons and encodes an 1863 amino acid protein, which contains multiple domains, each of them associated with one or more specific functions (Figure 1). The N-terminus is comprised of a zinc finger RING binding motif, which is normally present in DNA interacting proteins or proteins with ubiquitin ligase activity [10,11,12]. In fact, it has been demonstrated that BRCA1 when in complex with other proteins exhibits E3 ligase activity. However, this does not result in its substrates being targeted to the proteasome for degradation, but instead has a function in the DNA repair process [13,14]. BRCA1 also has two nuclear localization signals that direct its mobilization into the nucleus, where upon genotoxic stress it forms very distinct and punctuate nuclear foci [15]. Between the nuclear localization signals and the C-terminus of BRCA1 is a region that is not known to exhibit homology to any other proteins, but this domain is implicated in the interaction of BRCA1 with a number of proteins that function in the DNA repair process and the cell cycle checkpoint control. The C-terminal domain of BRCA1 contains two BRCT motifs in tandem that are present in other proteins known to have a function in DNA repair and DNA damage response [16,17]. As discussed below, the BRCT domains of BRCA1 mediate the interaction with signaling kinases and other proteins involved in cell cycle checkpoints. Both the RING and BRCT domains of BRCA1 are of utmost importance for normal BRCA1 function and it is not surprising that a large number of breast cancer predisposing mutations are located in both of these two domains (See Breast Cancer Information Core Data base [18]).

The formation of BRCA1 nuclear foci in S-phase synchronized cells, which are enhanced following genotoxic stress and co-localization of BRCA1 with the DNA repair protein RAD51, pointed to functions for BRCA1 in DNA repair processes and cell cycle checkpoint controls. Studies have revealed that BRCA1, together with a number of other proteins, plays an important function in safeguarding the integrity of the genome [19]. 

The actions of BRCA1 following DNA damage are dictated through its crosstalk with a network of proteins. Here we will review some of the best characterized interactions of BRCA1 and their functional implications. 

## 2. BRCA1 Interaction with the DNA Recombinase RAD51

BRCA1 demonstrates a pattern of nuclear foci formation in S-phase synchronized cells [15] which is very similar to that exhibited by the human recombinase RAD51 in phytohaemagglutinin stimulated lymphocytes [20]. This suggested that both proteins are involved in the double strand DNA damaged response pathway as RAD51 mediates HR and DNA strand exchange [21,22] and BRCA1-deficient cells demonstrated an impaired ability to repair DSBs via HR [8,23]. Scully and colleagues demonstrated that RAD51 and BRCA1 nuclear foci not only co-localize in S-phase synchronized cells, but that the there is a direct interaction between these two proteins [7]. Endogenous BRCA1 co-immunoprecipitates with RAD51 and this interaction is promoted via the region covering amino acids 758–1064 of BRCA1 [7]. However, the levels of Rad51 that co-precipitate with BRCA1 are low [7,24] and it is not entirely elucidated whether they form physical contacts or the association is mediated by other bridging proteins. The interaction of RAD51 with BRCA1 is implicated in the process of mitosis and meiosis, as the complex is visualized in the nuclei of unsynapsed axial complexes during the process of synaptonemal development [7]. BRCA2 also co-localizes with BRCA1-RAD51 complexes on mitotic and meiotic chromosomes after exposure to ionizing radiation (IR) or hydroxyurea [25]. Therefore, each of these proteins is involved in DNA repair via HR. It was further demonstrated that BRCA1 interacts independently with both BRCA2 and RAD51 [25,26,27,28,29], as the interaction with BRCA2 is mediated via the far C-terminus end of BRCA1 (residues 1314–1863) [25]. Nevertheless, it has not been shown yet with the use of recombinant proteins that BRCA1 forms direct contacts with either Rad51 or BRCA2. In addition, BRCA2 forms independent contacts with RAD51 through its BRC repeats [30,31,32,33,34] and C-terminal domains [35,36] and stimulates RAD51-mediated DNA strand exchange. Therefore, RAD51 does not act as a scaffold protein for the formation of a DNA repair complex. This is further supported by evidence that in BRCA2 deficient cells that express a functional BRCA1, HR by gene conversion predominates over single strand annealing indicating that BRCA1 activates RAD51 [37]. DNA damage stimulates the formation of BRCA1-RAD51 sub nuclear foci at stalled replication forks where BRCA1 and BRCA2 promote and stabilize the formation of nucleoprotein RAD51 filaments following DNA damage [38,39]. However, when either the function of RAD51 or BRCA2 is impaired, stalled replication forks are no longer stable and their degradation is promoted through MRE11 [39,40]. 

Therefore the formation of a complex between BRCA1 and RAD51 upon DNA damage, directs their mobilization at sites of DNA damage where BRCA1 and BRCA2 promote RAD51 nucleoprotein filament formation and stabilize RAD51 for the HR process. 

## 3. Phosphorylated BRCA1 Mediated Interactions and Their Function Cell Cycle Regulation

**Figure 2:**
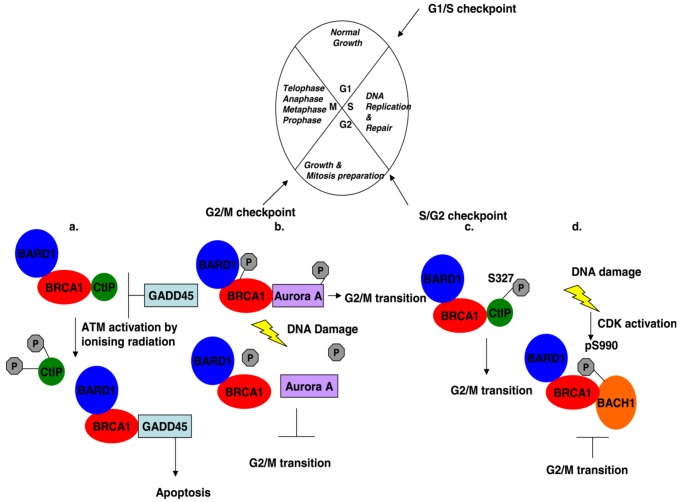
Cell cycle regulation mediated by BRCA1 and its associated proteins. (**a**) G2/M transition: Ionizing radiation induces phosphorylation of CtIP at residues S664 and 745, causing its dissociation from BRCA1, which represses GADD45 activation. Free BRCA1 activates GADD45 and downstream signaling leads to cell death by apoptosis instead of entering into mitosis. (**b**) The interaction of phosphorylated BRCA1 at serine residue 308 with phosphorylated Aurora A kinase regulates G2/M transition. DNA damage dephosphorylates both proteins and the complex dissociates with the effect that the cell gets stalled in the G2 phase. (**c**) BRCA1 controls transiently the G2/M transition through its interaction with phosphorylated CtIP at serine 327. (**d**) DNA damage activates Cyclin-dependent kinases in the G2 phase of the cell cycle, which phosphorylates BACH1 thus promoting the interaction with BRCA1 and cells accumulate in the G2 phase.

Following the discovery of the *BRCA1* gene and its linkage to breast and ovarian cancer, the BRCA1 protein was shown to be localized mainly to the nucleus, where it exhibits a dot pattern of expression in S phase synchronized cells [7,15,41], thus suggesting that this protein is involved in a cellular process taking place within the nucleus. This notion was further supported by the fact that the amino acid sequence of BRCA1 bears multiple serine and tyrosine residues that could be potential targets of phosphorylation which could be involved in transcription or protein kinase activation via phosphorylation events. Scully and colleagues were the first to demonstrate that BRCA1 is indeed phosphorylated; and that S-phase synchronized cells when treated with hydroxyurea not only exhibit DNA synthesis arrest and dispersal of the BRCA1 nuclear foci, but that BRCA1 becomes hyperphosphorylated [42]. In addition, they demonstrated that different phosphorylation events take place at different stages of the cell cycle [42] which has been subsequently supported by many investigators. A number of kinases are currently known to promote phosphorylation of BRCA1 at specific residues and in addition, they physically interact with BRCA1. Different kinases act at different cell cycle stages, thus implicating BRCA1 in cell cycle checkpoint regulation control. In this context, the checkpoint protein kinases Ataxia Telangiectasia mutated (ATM) [43,44], ATM-related kinase (ATR) [45] CHK2 (or CDS1) [46] and Aurora A [47] have been shown to phosphorylate BRCA1 at specific residues at different cell cycle checkpoints following genotoxic stress (Figure 2). It has been demonstrated that treatment with different DNA damaging agents promote the phosphorylation of specific BRCA1 residues and these events are implicated in cell cycle checkpoint controls [48]. In S-phase synchronized cells, IR induces the phosphorylation of BRCA1 at serines 988 and 1524, whereas UV induces phosphorylation at serine 988 [48]. Phosphorylation of BRCA1 at serine 988 affects its localization, as in the non-phosphorylated state the protein resides on the chromosome whilst IR- or UV-induced phosphorylation of serine 988 promotes the mobilization of the protein to the inner chromosomal structures of the centrosome and DNA replication forks [48]. However, different phosphorylation events take place in G2/M synchronized cells, where both IR and UV treatments promote phosphorylation of serine 1423 [48], thus suggesting that different kinases are involved at different cell cycle checkpoints. 

**Figure 3:**
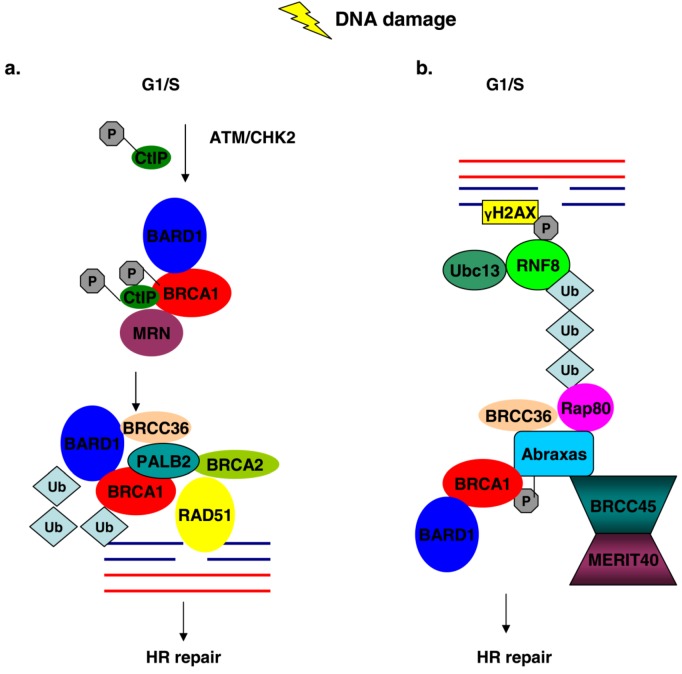
BRCA1 association with different protein complexes during the G1/S phase of the cell cycle regulates HR repair mechanisms in response to DNA damage. (**a**) DNA damage activates ATM and CHK2 kinases, which mediate phosphorylation of CtIP and BRCA1. Phosphorylated CtIP associates with BRCA1 and with the MRN complex leading to the recruitment of the BRCC complex at the site of DNA damage where HR is initiated. (**b**) Ubc13/RNF8 ligases are recruited at DNA lesions, where they facilitate the mobilization of Rap80-Abraxas-BRCA1 complex via the UIMs of Rap80, where ubiquitination events take place and promote HR process.

Initially it was demonstrated that ATM a well known tumor suppressor which belongs to the family of proteins related to phosphoinositide kinases, physically interacts with BRCA1 in irradiated cells [43,44], and that it specifically phosphorylates BRCA1 at serine residues 1423, 1524 and 1457 [43,44]. ATM is activated in response to DNA damage and is required for efficient DNA DSB repair and also for the phosphorylation and activation of other kinases, that promote apoptosis or cell cycle arrest (Figure 2 and Figure 3). BRCA1 co-precipitates with ATM [43,44] and this interaction is mediated between the BRCA1 amino acid sequence 1367 to 1395 and the N'-terminus of ATM as well as with domains of its C'-terminal domain [44]. Although there is an ATM dependency for BRCA1 phosphorylation, in A-T patient derived cells, which express a mutant inactive form of ATM, BRCA1 phosphorylation is not entirely ablated, but only delayed [44] suggesting that other kinases are involved in BRCA1 phosphorylation in S-phase synchronized irradiated cells. In light of this observation, it was soon revealed that ATR can also phosphorylate BRCA1 in an ATM-independent pathway in UV- or hydroxyurea-treated cells [45]. Serine 1423 is the main target of ATR mediated phosphorylation *in vivo*, but it was also demonstrated that other serine residues can also be phosphorylated *in vitro* [45]. Interestingly it was demonstrated that different DNA damaging agents can induce phosphorylation of serine 1423 residue via different pathways in S-phase synchronized cells. Specifically both UV and IR can induce either ATM or ATR mediated serine 1423 phosphorylation, with IR-treated cells demonstrating a preference for ATM-mediated phosphorylation [45]. However, ATM is not present in nuclear foci in cells arrested in the S-phase following DNA damage, whereas ATR is present at stalled replication forks like BRCA1 [45]. Therefore, ATM may induce BRCA1 phosphorylation in response to DNA damage signals that may promote the mobilization of the phosphorylated BRCA1 at the sites of damaged DNA, whereas ATR-mediated phosphorylation of BRCA1 takes place at sites of DNA damage and repair. Furthermore, ATM-mediated phosphorylation of BRCA1 residues 1423 or 1524 has no effect on the BRCA1-mediated HR and suppression of non-HR DNA repair processes [49]. 

CHK2 protein kinase has also been shown to regulate BRCA1 phosphorylation at sites of DNA damage [46] which is in agreement with the observation that BRCA1 is phosphorylated at nuclear foci and then disperses following DNA damage [42]. It is also compatible with the fact that the yeast homologue of CHK2, Cds1 protein kinase regulates the DNA damage response and replication checkpoint [50,51]. CHK2 protein kinase co-localizes with BRCA1 at nuclear foci and directly interacts with BRCA1 [46]. This interaction is dependent on CHK2 mediated phosphorylation of serine residue 988 of BRCA1, since following IR and BRCA1 phosphorylation, the complex is dissociated [46]. This was shown to be vital for the survival of HCC1937 cells that express a mutant truncated form of BRCA1 [52], after DNA damage [46]. Subsequently it was demonstrated that CHK2-mediated phosphorylation of BRCA1 is important for BRCA1-mediated HR, as cells expressing a S988A BRCA1 mutant or a defective CHK2 kinase fail to promote DNA repair via HR and instead non-HR events take place [49]. 

The interaction of BRCA1 with Aurora A and phosphorylation of BRCA1 have been demonstrated to regulate G2/M cell cycle transition [47]. Aurora A is a kinase known for its involvement in the regulation of cells entering into mitosis and its activation is inhibited by DNA damage [53,54]. The physical interaction of BRCA1 with Aurora A has been mapped to the C'-terminal domain of BRCA1. Aurora A gains its kinase activity through phosphorylation of its threonine residue 308 and active kinase promotes the phosphorylation of serine 308 in BRCA1 during the M phase (Figure 2). However, as cells gradually enter into mitosis BRCA1 serine 308 phosphorylation levels decrease [47]. Aurora A phosphorylation is impaired upon DNA damage, hence the kinase loses its activity and BRCA1 phosphorylation is inhibited, resulting in failure of the cells to proceed from G2 to mitosis [47]. 

It can be postulated that phosphorylation of BRCA1 at specific amino acid residues during different cell cycle stages, ensures genomic stability through the regulation of the cell cycle or promotion of DNA repair mechanisms. 

## 4. The BRCC Complex of Proteins

The identification of BRCA1 and BRCA2 proteins and their association to breast cancer provoked numerous studies aiming to identify potential ligands of these proteins and decipher their function. BRCA1-associated RING domain (BARD1) protein was one of the first proteins identified to interact with BRCA1 [55]. The RING domain of BRCA1 was used as bait in yeast two hybrid experiments with a B cell line library acting as prey and revealed that BARD1 associated with BRCA1. This interaction was also confirmed *in vivo* and BARD1 characterization revealed that it contains a cysteine-rich domain in its N-terminus which matches to the RING motif, three tandem ankyrin repeats and a C-terminal domain which shows high sequence homology to the conserved BRCA1 BRCT domain [55]. Further characterization of the interaction between the two proteins, revealed that the interaction is not wholly dependent on the RING motif of either protein, but instead the interaction depends on a larger structural domain, with minimal requirement the amino acid sequence 1–109 of BRCA1 and residues 26–119 of BARD1 [56]. The presence of a RING domain on both proteins and the finding that the association of the heterodimer is through the RING domain would suggest that the heterodimer associates with nucleic acids. However, it was shown that the heterodimer has no affinity for DNA [56], thus suggesting that the heterodimer may act as a scaffold for other nucleic acid binding proteins or that it has other functions. However, the discovery that the small RING finger proteins ROC1 and ROC2 are ubiquitin ligases [57,58,59], prompted scientists to investigate whether the RING associated BRCA1-BARD1 heterodimer is an ubiquitin ligase itself. Hashizume and colleagues were the first to report that the heterodimer is indeed a ubiquitin ligase and more specifically that it promotes polyubiquitin chain polymerization in collaboration with the E2 ubiquitin UbcH5c [13]. The E3 ligase activity of the heterodimer is not affected by mutations in BARD1, whereas the cancer associated C61G missense mutation, which removes the conserved zinc-binding cysteine from the RING domain of BRCA1 completely abolishes the ubiquitin ligase activity of the heterodimer [13,60]. However, BRCA1 association with BARD1 is essential for the E3 ligase activity of BRCA1 and the interaction stabilizes both proteins and most of BRCA1 is usually in complex with BARD1 under normal conditions [13,60]. In addition unlike with other proteins, the polyubiquitinated heterodimer or its substrates are not targeted to the 26 S proteasome for degradation. In addition, the heterodimer has the ability to autoubiquitinate itself which further enhances its E3 ligase activity [14]. The E3 ligase activity of the heterodimer was confirmed by others who further demonstrated that specific substrates for ubiquitination is the histone protein H2A [14,60] and the nucleosome core histones H2B, H3 and H4, but not the linker histone H1 [14]. In addition, the general target for ubiquitination by the E3 ligases, the denatured lysozyme does not serve as a substrate for the BRCA1-BARD1 heterodimer [14], thus supporting the notion that this E3 ligase activity of the heterodimer is not linked to protein degradation but instead has a different function. 

Ubiquitin has seven lysine residues, which are potential sites for polyubiquitin chain attachment [61] with chains attached on the K48 and K29 residues being targeted to the proteasome for degradation, whereas K63-linked chains do no target proteins for destruction, but instead signal for regulatory pathways such as DNA repair [62]. Even though the BRCA1-BARD1 heterodimer can catalyze the formation of K48 and K63 ubiquitin linked chains [60,63], it shows preference for the formation of the unusual K6-linked chains and BRCA1 is autoubiquitinated with K6-linked polymers [63,64]. Unlike K48-linked chains which are targeted for degradation by the 26S proteasome, BRCA1-BARD1 heterodimer with K6-linked ubiquitin chains are not degraded but can be de-ubiquitinated *in vitro* [64]. The preference of the heterodimer for K6-linked polyubiquitin chains was confirmed, but it was also demonstrated that in K6R ubiquitin transfected cells, the formation of BRCA1 and γH2AX foci is intact, thus suggesting that the E3 ligase activity of the heterodimer is downstream the formation of BRCA1 and γH2AX foci [65]. 

The BRCA1-BARD1 heterodimer was found to be a part of a larger complex of proteins called the BRCA1-BRCA2- containing complex (BRCC) [24]. Investigators used epitope tagged-BARD1 protein in order to isolate possible new partners of the BRCA1-BARD1 heterodimer in nuclear extracts of 293T and H1299 cells. BRCA1 was found to be forming a complex with BRCA2, RAD51 and BRCC36 and BRCC45 [24]. BRCC36, a protein which has sequence homology with the Poh1/Pad1 of the 26S proteasome and with the subunit 5 of the COP9 signalosome was shown to interact with BRCA1 [24]. However, it remains unclear whether the two proteins form physical contacts. Mutations that abolish the interaction between BRCA1 and BRCC36 reduce the association of BRCA2 and RAD51 to the complex, whereas mutations that abolish BARD1 interaction with BRCA1 had no effect on the association of the above proteins [24]. These findings suggest that BRCC36 may be acting as a scaffold protein bringing other partners to the complex, whereas BARD1 does not have a structural function. BRCC36 was later shown to have a de-ubiquitinating activity with a preference for K63-linked polymers [66] and therefore its function is not limited to being a scaffold protein, as will be discussed later. The BRCC complex was shown to have an Ubc5-dependent E3 ligase activity which is in agreement with other studies. 

The function of the BRCA1-BARD1 heterodimer at cell cycle checkpoints was investigated since it has been extensively demonstrated that BRCA1 expression levels increase in late G1/S phase of the cell cycle where it forms nuclear foci at sites of DNA damage [7,15,25] and that DNA damaging agents such as hydroxyurea or IR stimulate the recruitment of BRCA1 at sites of DNA damage and HR [42,67,68]. Morris and colleagues demonstrated that in S-phase but not in G1 synchronized cells, BRCA1 formed nuclear foci which co-localized with conjugated ubiquitin and BARD1 [65]. Introduction of DSBs either by treating cells with hydroxyurea or exposure to IR, caused an enhanced accumulation of conjugated ubiquitin foci which co-localized with BRCA1 and γH2AX, thus suggesting that the heterodimer is active as an E3 ligase at sites of DNA damage. The requirement for an intact BRCA1-BARD1 complex for ubiquitination events at sites of damaged DNA, was further supported as siRNA mediated depletion of BRCA1 inhibited the formation of conjugated ubiquitin foci and also caused reduced BARD1 expression [65]. Furthermore it was demonstrated that over expression of both BRCA1 and BARD1 enhances the formation of conjugated ubiquitin foci in IR treated cells, and the E3 ligase activity of the heterodimer requires an intact RING finger domain in BRCA1 [65].

Furthermore, it has been demonstrated in mouse models of familial breast cancer that the I26A mutation in the RING domain of BRCA1 that does not disrupt its heterodimerization with BARD1, but abolishes its E3 ligase activity firstly does not cause embryonic lethality and animals grow to normal adulthood, although male animals are sterile; and secondly the rate of spontaneous tumor formation is the same as in animals expressing wild type BRCA1 thus suggesting that it is not the E3 ligase activity of BRCA1 that confers its tumor suppression function [69]. The authors suggested that the tumor causing mutations in the RING domain of BRCA1 are the missense mutations that abolish BRCA1-BARD1 heterodimerization and not the ones that abolish the E3 ligase activity of BRCA1 [69]. Therefore, BARD1 functions in stabilizing BRCA1 as well.

The exact function of the BRCC complex in HR is not entirely elucidated, but its E3 ligase activity may promote the mobilization and stabilization of the complex at broken chromatin sites were the complex can stabilize the formation of RAD51 filaments thus facilitating the repair process (Figure 3a). 

## 5. PALB2 Acts as Bridge between BRCA1 and BRCA2

PALB2, a nuclear protein was initially identified as a binding partner of BRCA2 and was demonstrated to co-localize with both BRCA2 and BRCA1 in S-phase synchronized cells [70]. It was later demonstrated that PALB2 directly interacts with BRCA1 as well [71,72,73]. Sy and colleagues using tandem affinity purification demonstrated that PALB2 co-precipitated with two larger proteins [71]. Analysis by mass spectrometry confirmed that the one protein was BRCA2 as expected and revealed that the second band corresponded to BRCA1 [71]. The interaction was confirmed *in vivo*, as endogenous BRCA1 co-precipitated PALB2 [71]. In addition, the direct binding of BRCA1 with PALB2 was demonstrated using recombinant BRCA1 protein (residues 1293 to 1863) fused to GST, to pull-down exogenously expressed PALB2 in EUFA1341 fibroblasts [70]. The physical interaction between BRCA1 and PALB2 has been mapped on the coiled-coil domain in the amino terminus of PALB2 and residues 1367–1437 of BRCA1 which fall within its coiled-coil domain as well [71,72,73]. The interaction of BRCA1 with PALB2 is disrupted upon deletion of the amino terminal domain coiled-coil domain of PALB2 [71,73] or L21P and L24P mutations in this domain [72]. However, these mutations do not abolish the binding of BRCA2 with PALB2 [72]. 

The finding that PALB2 interacts with both BRCA1 and BRCA2 prompted many groups to investigate whether all three proteins exist in a complex under native conditions. Gel filtration chromatography and western blot analysis of nuclease treated 293T cell lysates, demonstrated that PALB2 co-eluted with BRCA1 and BRCA2 and that the DNA repair machinery proteins CtIP, BACH1, Rad51 and RPA were also present in the same fraction [71]. As the region in BRCA1 that mediates the interaction with PALB2 overlaps with the region that has been reported to mediate interaction with BRCA2 [25] it was speculated and then demonstrated that PALB2 forms a bridge to link BRCA1 and BRCA2 as BRCA2 fails to co-precipitate with BRCA1 in siRNA mediated PALB2 depleted cells [71]. In addition, it was demonstrated that in cells expressing a PALB2 mutant that abolishes its interaction with BRCA1, PALB2 maintains its ability to interact with BRCA2, but BRCA2 does not co-precipitate with BRCA1 [71]. 

In light of the ability of PALB2 to act as a scaffold protein linking BRCA1 with BRCA2 and that these proteins co-elute with other DNA repair proteins sparked speculations that this complex might be involved in HR. Many investigators demonstrated that in irradiated cells, BRCA1 and BRCA2 nuclear foci co-localize with PALB2 [71,72,73] and that PALB2 also co-localizes with γH2AX [70,71] and Rad51 [71], further supporting the evidence that PALB2 is a chromatin associated protein, as it is extracted from lysed cells under high salt buffer conditions [71,73]. BRCA1 mobilization at sites of DNA lesions following IR is not entirely dependent on PALB2, since BRCA1 foci are still formed in PALB2 depleted or mutant cells, although some reduction is observed [71]. However, BRCA2 recruitment at DSBs is impaired in PALB2 depleted cells or BRCA1 mutant cells where BRCA1-PALB2 interaction is abolished, suggesting that an intact interaction between BRCA1 and PALB2 is important for targeting BRCA2 at DNA lesions [71,73]. 

The interaction of BRCA1 with PALB2 is believed to be involved in the HR repair process, because cells expressing a PALB2 deletion mutant, which abolishes interaction with BRCA1, are hypersensitive to mitomycin C treatment [71]. In addition, it has been demonstrated by gene conversion and reporter assays that PALB2 depletion or expression of deletion mutant that abolishes BRCA1 interaction, causes HR suppression and re-introduction of PALB2 reverses the effects [71,73]. 

## 6. BRCA1 Associates with the Mrn Complex during the Late S/G2 Phase of the Cell Cycle

RAD50, another DNA recombinase forms a complex with MRE11 and NBS1 (also known as p95/nibrin) known as MRN and this complex of proteins is implicated in the DNA damage response pathway via both HR and non-homologous end-joining (NHEJ) and in telomere maintenance [74]. Surprisingly BRCA1 also associates with the MRN complex of proteins [67,68,75] and this association is cell cycle dependent and peaks in late S and G2 phases [68]. Initially it was demonstrated that the association of BRCA1 with the MRN complex was mediated via direct interaction of RAD50 amino terminal domain with residues 341–748 of BRCA1 [68] but very soon it was shown that that the other members of the MRN complex can also co-immunoprecipitate with BRCA1 [67]. The BRCA1-MRN complex does not dissociate upon treatment with IR, it remains intact [68] and is actually induced as BRCA1 foci exhibit a strong co-localization pattern with MRN in S-phase synchronized cells following treatment [67]. This has also been demonstrated by immunoprecipitation experiments, where stable cell lines expressing dual tagged BARD1 (FLAG/HA) were generated [76]. Following IR treatment and FLAG/HA affinity purification the presence of BRCA1-BARD1-MRN complex was enhanced in the immunoprecipitates [76]. Furthermore, following IR treatment, BRCA1 is in a hyperphosphorylated state [68], possibly via ATM/ATR dependent phosphorylation [45]. Greenberg and colleagues not only confirmed the partial dependency of ATM-mediated phosphorylation of BRCA1 and interaction with the MRN complex, but they further demonstrated that this process is also dependent on CHK2 activation (Figure 3a) [76].

The BRCA1-MRN complex exhibits two distinct patterns of localization after IR, which is dependent on the cell cycle phase [67]. In G1 arrested cells following IR, BRCA1 foci are barely detectable, whereas there are bright and distinct nuclear foci of the MRN complex. In contrast, in S phase arrested cells, BRCA1 forms bright and distinct foci, whereas the MRN complex exhibits a more diffused nuclear localization pattern, which co-localizes with a number of BRCA1 foci [67]. 

S-phase irradiated cells that exhibit BRCA1-MRN foci co-localization do not simultaneously form BRCA1-RAD51 [67,68], nor BRCA1-BRCA2-RAD51 foci [67]. The mutual exclusive association of BRCA1 with the MRN and BRCC complexes in S-phase irradiated cells, suggests that the former complex may act as a sensor of DSBs and signals for the recruitment of the downstream DNA repair machinery including BRCC mobilization at the sites of damaged DNA, whereas the BRCC complex has a direct function in the HR process (Figure 3a).

## 7. The Interaction of BRCA1 with CtIP at Different Stages of the Cell Cycle Has Specific Functions

Yeast two-hybrid ligand fishing experiments identified CtIP as a BRCA1 interacting protein and the interaction is mediated through the two C'-terminal BRCT domains of BRCA1 [77,78]. The interaction of BRCA1 with CtIP has multiple functions, depending on the cell cycle stage, other interacting partners, on the phosphorylation status and ubiquitination events.

The interaction of BRCA1 with CtIP was initially implicated in the G2/M checkpoint and apoptosis regulation. This interaction depends on the phosphorylation status of CtIP, as IR induces the ATM-mediated hyperphosphorylation of serine residues 664 and 745 of CtIP, which results in the dissociation of CtIP from BRCA1 [79]. CtIP interaction with BRCA1 inhibits BRCA1 from inducing *GADD45* gene expression and activation, which also functions in the DNA damage response pathway [79]. BRCA1-mediated stimulation of GADD45 expression promotes GADD45 interaction with the MTK1/MEKK4 MAPKK kinases [80], which then phosphorylate the JNK/SAPK kinases leading to cell apoptosis [81]. In addition, BRCA1 induces the phosphorylation of MEKK4 and JNK in stressed cells leading to cell death by apoptosis [82]. However, BRCA1 is further implicated in the induction of apoptosis via its interaction with MEKK3, where it possibly acts as a scaffold to bring together mediators of the MAP3K cascade [83]. Therefore, BRCA1 through its association with CtIP is implicated in the induction of apoptosis following genotoxic stress (Figure 2). 

During the G2/M cell cycle checkpoint, CtIP serves as a substrate for ubiquitination by the BRCA1-BARD1 heterodimer when serine 327 is phosphorylated [84]. In cells treated with genotoxic agents, BRCA1 mediated ubiquitination of CtIP does not target CtIP in the proteasome for degradation, but instead it is targeted to the sites of DNA damage where it associates with chromatin and co-localizes with γH2AX and BRCA1 [84]. BRCA1 mediated ubiquitination of CtIP, is dependent on the phosphorylation status of the serine 327 residue, as its interaction with BRCA1 is abrogated when this residue is mutated to alanine [79,85] and is not ubiquitinated by the BRCA1-BARD1 heterodimer [84]. 

**Figure 4:**
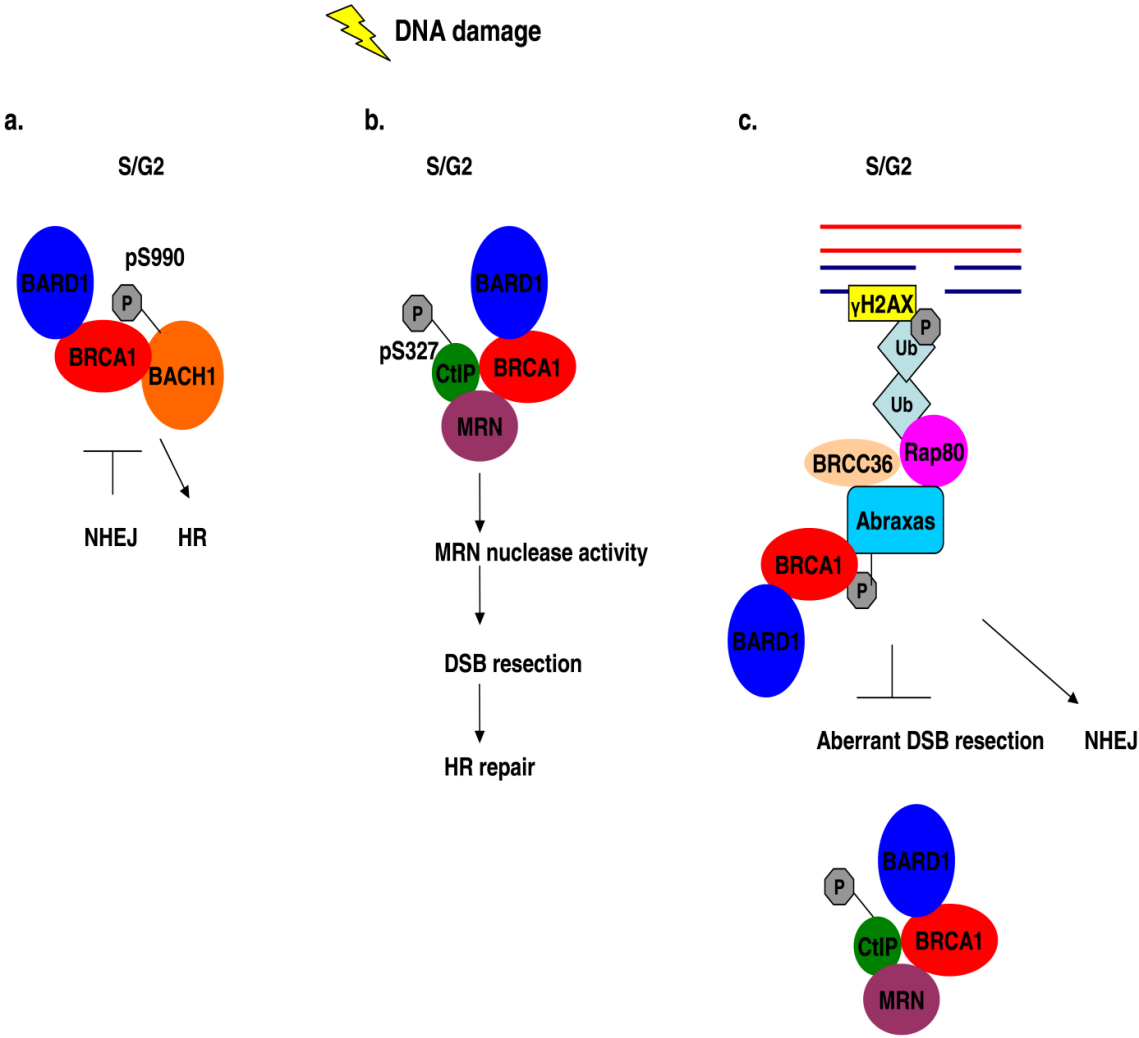
BRCA1 association with different protein complexes during the S/G2 phase of the cell cycle regulates HR repair mechanisms in response to DNA damage. (**a**) The error-prone NHEJ is inhibited by the interaction of phosphorylated BACH1 with BRCA1 in S/G2 damaged cells and HR is promoted instead. (**b**) DNA damage in S/G2 cells induces the association of phosphorylated CtIP with BRCA1 and MRN, thus enhancing the nuclease activity of the MRN complex of proteins which enables efficient HR. (**c**) The interaction of Rap80 with BRCA1 in S/G2 cells following DNA damage blocks aberrant HR events which can be deleterious.

The interaction of BRCA1 with CtIP, in which serine 327 is phosphorylated, is possibly involved in the G2/M DNA damage checkpoint control, where it may regulate the activation of CHK1 [85] but also in the S/G2 checkpoint control, where its interaction with BRCA1 and the MRN complex enhances the nuclease activity of MRN. The latter promotes DSB resection, which facilitates DNA repair via HR and blocks NHEJ, which normally takes place in G1 arrested cells and is compromised in CtIP negative cells (Figure 4b) [86,87]. 

The association of BRCA1 with CtIP is implicated not only in promoting HR over the error prone NHEJ when MRN is also part of the complex, but facilitating HR through ubiquitination events as well. However, dissociation of the complex in the G2/M checkpoint promotes apoptosis in damaged cells. 

## 8. BRCA1 Forms a Complex with Phosphorylated BACH1 (BRIP1)

The interaction of BRCA1 with BACH1 was identified and characterized by Cantor and colleagues [88]. The N-terminal domain of BACH1 resembles that of the family of DEAH helicases, as it contains seven helicase-specific motifs and a nuclear localization signal, and the interaction with BRCA1 is mediated through its C-terminal domain; and the BRCT of BRCA1 [88,89]. The crystal structure of the BRCT domains of BRCA1 in complex with the phosphorylated N-terminal domain of BACH1 demonstrated that the BRCT domains exhibit only local conformational changes when in complex with BACH1 [90]. The phosphorylated residues serine 990 and phenylalanine 993 of BACH1 mediate the contact by burying themselves within the cleft created by the stacking of the BRCT domains, thus explaining why BRCT mutants on the cleft disrupt the interaction [90]. Like with CtIP, the association of BACH1 with BRCA1 is dependent on the phosphorylation status of BACH1 as in the presence of phosphatase inhibitors the interaction between BACH1 and BRCA1 is increased, whereas in the presence of λ phosphatase the interaction is abolished [89]. In addition, it was further demonstrated *in vivo* that the association is dependent on serine 990 phosphorylation of BACH1 [89] and the crystal structure of the complex further confirmed the requirement of BACH1 phosphorylation.

BACH1 protein expression levels remain unchanged throughout the cell cycle [89]. However, during the S and G2 phase of the cell cycle BACH1 forms punctuate nuclear foci which co-localize with BRCA1 foci [88,89] and phosphorylation of serine 990 on BACH1 takes place only in the S and G2/M phases of the cell cycle and not in G1 synchronized cells [89]. These observations together with the presence of a proline residue next to the serine 990, suggest that BACH1 phosphorylation is cyclin-dependent kinase regulated and it is involved in cell cycle checkpoint control like BRCA1 (Figure 2), as it has been demonstrated that siRNA mediated BACH1 depletion impairs G2 arrest and cells accumulate in the M phase [89]. 

The interaction of BRCA1 with BACH1 is not only implicated in the G2/M checkpoint control, but possibly plays a role in double strand DNA repair, as mutations in the helicase domain of BACH1 which impair the interaction with BRCA1, disrupt DSB repair via the activation of HR [88,91] and it has been suggested that this interaction ensures that NHEJ process is suppressed by BRCA1 and HR is activated instead (Figure 4a) [91].

This is further substantiated in a mouse model of familial breast cancer described by Shakya and colleagues that generated mice with a BRCA1^S1598F/S1598F^ background, a mutation that disrupts the interaction of BRCA1's BRCT domain with BACH1 [69]. These mice are sterile but show normal development. However, mouse embryonic fibroblast cells derived from these mice demonstrate proliferation defects, chromosomal instability and impaired recruitment of repair proteins at sites of DNA damage [69]. In addition, embryonic stem cells isolated from these animals are hypersensitive to treatment with mitomycin C and show defects in HR process [69]. 

## 9. BRCA1 Interacts with the Phosphoproteins Abraxas (CCDC98) and Rap80

In search of phosphorylated proteins that bind BRCA1 BRCT domains, Abraxas and Rap80 were identified as novel BRCA1 partners and the interaction with phosphorylated BACH1 and CtIP was further confirmed [92,93] There is a specific requirement for phosphorylation of residues serine 404 and 406 or 406 only for interaction with BRCA1. Phosphorylated Abraxas in the above mentioned residues but not on 404 only is enriched in the nuclei of IR treated cells [93]. Rap80 which is a substrate for ATM/ATR mediated phosphorylation following IR treatment [94,95,96], has been identified to associate with the BRCT domain of BRCA1 [93,94,95] and the interaction of Abraxas and Rap80 with BRCA1 is mutually exclusive with BACH1 and CtIP [93]. In addition in the Abraxas-Rap80-BRCA1 complex, BARD1 is also part of it, suggesting that BRCA1:BARD1 may exhibit E3 ligase activity on Rap80 which contains two ubiquitin interacting motifs [93] and shows a preference for K63 and K6-linked ubiquitin chains [95]. BRCC36 is also part of this complex and associates with both Rap80 and Abraxas [97]. BRCC36 may control the aberrant ubiquitination events by BRCA1 through its de-ubiquinating activity. Furthermore, it has been demonstrated that in M-phase cells Rap80 exists in complex with the BRCA1-BARD1-BRCC36-BRCC45 complex which is very abundant and its association is dependent on BRCC36 [95]. It was later then demonstrated that BRCA1-Abraxas-Rap80-BRCC36 are part of a much larger complex of proteins following DNA damage [98]. In search of more partners of the above complex Feng and colleagues generated 293T cell lines expressing triple tagged Rap80, Abraxas or BRCC36 [98]. In the proteomic analysis they performed they identified and confirmed that BRCC45 (BRE) and MERIT40 (Hspc142) are also part of this complex and that this heterodimer is responsible for the integrity of the Abraxas-Rap80-BRCC36 complex [98]. It was demonstrated that MERIT40 strongly associates with BRCC45 offering stability to it; and that BRCC45 is the protein that bridges the interaction between MERIT40 and Abraxas-Rap80-BRCC36 [98]. However, in this complex, Abraxas forms contacts with BRCC36 and BRCC45 via different regions [98].

Cells depleted of Abraxas and Rap80 are hypersensitive to IR and UV treatment and exhibit defects in both the G2/M checkpoint and HR, although these defects are not as severe as those caused by BRCA1 depletion [93,94]. Abraxas and Rap80 form distinct nuclear foci in irradiated cells synchronized in the G1/S phase and co-localize with BRCA1 foci [93,94,95]. In addition, it has been demonstrated that the entire BRCA1-Abraxas-Rap80-BRCC45-BRCC36-MERIT40 co-localizes with γH2AX foci following IR [98]. Even though Abraxas-Rap80 foci formation is independent of BRCA1, depletion of Rap80 or mutations that destroy its ubiquitin interacting motifs (UIMs), cause a reduction in the BRCA1 foci formation, thus demonstrating a dependency of BRCA1 activity on Rap80 [93,94,95]. Recently new mutations in Abraxas and Rap80 have been identified which impair BRCA1 mobilization at sites of DNA damage [99,100]. In IR-treated cells Rap80 foci co-localize not only with BRCA1, but also with γH2AX and MDC1 [94,95] and Rap80 associates with chromatin only after DNA damage [94]. The mobilization of Rap80 to chromatin and DSBs is dependent on the presence of phosphorylated γH2AX and MDC1 and on its intact UIMs [94,95]. This suggests that Rap80 gains access to sites of DNA damage through its UIMs and acts upstream of BRCA1 and that this complex of proteins recruits BRCA1 at sites of DNA damage. More light as to the mechanism of Rap80 recruitment at sites of DNA damage was shed by investigators who demonstrated that the E2 ubiquitin ligase Ubc13 and the E3 ligase RNF8 are required for Rap80-Abraxas-BRCA1 mobilization at DSBs and polyubiquitin foci formation following IR [97]. RNF8 contains a phospho-amino acid interacting motif (FHA) which is necessary for IR induced foci, possibly through interaction with other phosphorylated proteins [97]. These observations, along with the Rap80 dependence on the presence of phopshorylated γH2AX and MDC1 at DSBs for its recruitment, prompted the investigators to suggest that IR induces the recruitment and phosphorylation of γH2AX by the ATM/ATR kinases at DNA lesions, which then recruits MDC1 where it becomes phosphorylated [97]. In turn, the phosphorylated MDC1 serves as a binding substrate for Ubc13/RNF8 through the FHA domain of RNF8. Then, RNF8 which recognizes K63-linked ubiquitin chains promotes the recruitment of Rap80-Abraxas-BRCA1 at the DSB through the UIMs of Rap80 (Figure 3b) [97].

Morris and colleagues further demonstrated that in cells treated with genotoxic agents (IR, cisplatin, and hydroxyurea) SUMO (small ubiquitin modifier) also co-localizes with γH2AX and BRCA1 [101]. In addition, it has been shown that SUMO physically associates with BRCA1 and promotes its sumoylation, which actually enhances the E3 ligase activity of BRCA1-BARD1 heterodimer [101]. BRCA1 modification by addition of sumo conjugates was shown to be mediated by PIAS E3 SUMO ligases and this process is required for DSB DNA repair [101]. Recently it was reported that not only BRCA1 is associated with SUMO, but that Rap80 contains a sumo interacting motif (SIM) which is in tandem with its two UIMs [102]. The SIM of Rap80 binds specifically to SUMO2/3 conjugates and proteomic analysis of GST-tagged SUMO2 pull-downs, demonstrated that BRCA1-Abraxas-Rap80-BRCC45-BRCC36 and BRCA1 are present [102]. The Rap80 dependency for sumoylation of the complex was shown, as siRNA mediated Rap80 depletion abolishes the interaction of the complex with SUMO2 [102] and it was further shown that both SIM and UIMs of Rap80 are required for the recruitment of Rap80 at DNA lesions [102]. However, it still remains to be elucidated how these modifications confer resistance to genotoxic stress. The partial dependency of BRCA1 mobilization to sites of DSBs on Rap80 and Abraxas, suggests that Rap80 is responsible for the recruitment of BRCA1 at sites of DNA damage, where BRCA1 sumoylation facilitates HR; and that attachment of SUMO and ubiquitin conjugates promotes the recruitment of Rap80 at DSBs which in turn facilitates BRCA1 recruitment and initiation of HR process.

However, the Rap80-BRCA1 complex has another function, where it limits HR during the S/G2 phase by associating with ubiquitinated chromatin structures at the DSBs [103]. This association can block the access to nucleases and more specifically to CtIP-MRN complex, thus protecting the site from uncontrolled end resection, which could be deleterious and lead to loss of genomic integrity (Figure 4c) [103]. The investigators therefore propose that the Rap80-BRCA1 complex influences the balance between HR and NHEJ [103]. 

## 10. BRCA1 Interaction with the Mismatch Repair Proteins MSH2, MSH3, MSH6 and MLH1

Wang and colleagues in their search for BRCA1 interacting partners, confirmed not only the association of BRCA1 with BARD1 and ATM, but also demonstrated the association of BRCA1 with the MRN complex, with BLM helicase and with the mismatch repair proteins MSH2, MSH3, MSH6 and MLH1 [67]. They named this entire complex BASC, from BRCA1-associated genome surveillance complex. The interaction of BRCA1 with the mismatch repair proteins implicates BRCA1 in transcription-coupled repair process, which may explain the interaction of BRCA1 with the RNA polymerase [104]. BRCA1 can associate with MSH2 via three independent regions: its RING domain (1–175aa), the region covering the amino acids 303–625 and its BRCT domains (773–1863). Interestingly BRCA1 contacts MSH2 on its C-terminal region [87], which is responsible for transducing the mismatch/lesion binding signal effectors through its ANB domain [105,106]. However, BRCA1 interacts only with the nucleotide free or ATP-bound and not with the ADP-bound form of MSH2-MSH6 heterodimer, thus indicating that this complex is actively involved in ATP hydrolysis in nucleotide mismatched repair regions [87]. This is further supported by the observation that this interaction is visualized in the nucleus of cells treated with the DNA damaging agent cisplatin [87]. 

## 11. Conclusions

BRCA1 has a pivotal role in maintaining genomic integrity as it is actively involved not only in DNA repair mechanisms, but also it is a caretaker of the normal cell cycle. The charisma of BRCA1 to be involved in multiple processes during distinct cell cycle stages is attributed to its ability to form different protein complexes, each of which has a specific function. However, the exact mechanism of action for many of these complexes is not entirely elucidated and this area requires further investigation. However, some complexes have similar functions and they are mutually exclusive, thus suggesting that the ability of BRCA1 to interact with multiple different complexes is a degenerative mechanism of the cellular machinery to ensure that BRCA1 will be able to perform its functions even when one or more of its partners are absent. In addition this suggests that the formation of one complex has an upstream role of another complex and possibly promotes the formation of downstream BRCA1-containing complexes which will ensure activation of HR. BRCA1 loss in mice leads to embryonic lethality (mouse models reviewed in [107]), but it has been shown that it can be rescued by concomitant loss of 53BP1 [108]. These mice develop to normal adulthood, although male mice are sterile and spontaneous tumorigenesis has the same frequency as wild type mice [108,109]. In addition, the HR process in these animals and in ES and MEF derived cells is not defective [109,110,111] as the deletion of *53BP1* gene promotes ATM-dependent DNA resection [109,110]. 

To conclude, the BRCA1 protein is a key player in DNA repair processes and normal cell cycle, which is further supported by the fact that mutations that disrupt the association of BRCA1 with one or more of its interacting partners are associated with increased risk for developing breast and ovarian cancer. A complete understanding of the exact mechanism of action of each complex is required, in order to proceed and apply targeted therapies in breast and ovarian cancer patients. 
